# Insufficient Sleep and Behavioral Health in the Military: A 5-Country Perspective

**DOI:** 10.1007/s11920-024-01497-1

**Published:** 2024-05-03

**Authors:** Sara E. Alger, Clare Bennett, Neanne Bennett, Matthew G. Huebner, Jennifer E. C. Lee, Heather J. McCuaig Edge, Amos Simms, Amy B. Adler

**Affiliations:** 1 Center for Military Psychiatry and Neuroscience, Walter Reed Army Institute of Research, 503 Robert Grant Ave, Silver Spring, MD 29010 US; 2 Defence Health Directorate, New Zealand Defence Force, Wellington, New Zealand; 3 Australian Defence Force, Joint Health Command, Canberra, Australia; 4 Director General Military Personnel Research and Analysis, Department of National Defence, Ottawa, Canada; 5 Academic Department of Military Mental Health, King’s College London, London, UK

**Keywords:** Sleep loss, Behavioral health, Leadership, Military, International

## Abstract

**Purpose of Review:**

The goal of this paper was to highlight the degree to which sleep, behavioral health, and leader involvement were interrelated using data from militaries in five English-speaking countries: Australia, Canada, New Zealand, the UK, and the United States.

**Recent Findings:**

Many service members reported sleeping fewer than the recommended 7 h/night: 34.9%, 67.2%, and 77.2% of respondents from New Zealand, Canada, and the United States, respectively. Countries reporting shorter sleep duration also reported fewer insomnia-related difficulties, likely reflecting higher sleep pressure from chronic sleep loss. Across all countries, sleep problems were positively correlated with behavioral health symptoms. Importantly, leader promotion of healthy sleep was positively correlated with more sleep and negatively correlated with sleep problems and behavioral health symptoms.

**Summary:**

Insufficient sleep in the military is ubiquitous, with serious implications for the behavioral health and functioning of service members. Leaders should attend to these risks and examine ways to promote healthy sleep in service members.

## Introduction

Despite technological advances in military operations, the nature of these operations remains a human endeavor, with service members at the core. Thus, the ability of service members to obtain adequate sleep, defined as 7–9 h in a 24-h period [1], is essential given that healthy sleep underlies the ability of service members to function cognitively, physically, and emotionally. However, military stressors, the physical environment, and poor sleep hygiene are factors that degrade service member sleep.

Without adequate sleep, service member health, performance, and, ultimately, the mission may be compromised. Sleep loss alters brain activity and connectivity in regions such as the prefrontal cortex and medial temporal lobe, impairing the ability to make decisions quickly and accurately [2], exercise good judgement [3], make and store new memories [4], attend and react to threat [5], communicate effectively [6], solve problems effectively [3], and think innovatively [7]. Collectively, these cognitive impairments can degrade performance, which, in turn, puts individuals and their team at risk.

Lack of sufficient sleep on a regular basis also risks impairing physical health, including greater likelihood of musculoskeletal injury, reduced muscle strength, diminished muscle repair [8], and decreased endurance [9]. In the short term, testosterone has been found to be reduced by 25–30% after 24 h of sleep deprivation [10]. In the long term, regularly restricting sleep is associated with an increased risk of chronic diseases such as obesity, type 2 diabetes, cancer, heart disease, stroke, and hypertension [11, 12].

Sleep loss is also linked to an impaired ability to regulate emotional and reward areas of the brain, leaving them unchecked and overactivated [13•, 14]. This dysregulation results in increased negative mood and bias to perceive the world more negatively and an increased stress response [15, 16]. Ultimately, there is greater risk for behavioral health problems, an umbrella term that generally refers to mental health problems, substance use disorders, and stress-related symptoms. By impairing emotion regulation, inadequate sleep may negatively impact behavioral health, potentially resulting in depression, anxiety, and post-traumatic stress disorder (PTSD) symptoms [15, 35–40].

Considering these deleterious consequences of sleep loss, military leaders have begun to address changing military culture surrounding sleep. Historically, powering through sleep loss was practically a badge of honor, a symbol of toughness and dedication to duty while succumbing to fatigue was seen as weak and lazy. There has since been a shift toward creating a culture that emphasizes the importance of sleep. This shift is supported through educating leaders about sleep and encouraging leaders to monitor their own sleep behavior and the sleep of their service members. In the United States (US), for example, initiatives, such as the US Army’s Performance Triad, have been launched to emphasize the value of sleep along with activity and nutrition “for achieving optimal physical, mental, and emotional health and wellbeing” [17]. In the Canadian Armed Forces, the Balance Strategy was created to enhance the “culture of fitness and improve operational effectiveness” through healthy behaviors that include sleep. Beyond health promotion strategies, military doctrine has also begun to emphasize the importance of service members obtaining adequate sleep. For example, the US Army Field Manual (FM) on Holistic Health and Fitness (FM 7-22) dedicated a chapter to sleep.

However, enacting real change in terms of the military culture’s perspective on sleep continues to be a challenge that is apparently international, with lack of sleep an epidemic in militaries across countries. The pervasive bias against sleep is reflected in statements such as “I’ll sleep when I’m dead” or “Sleep is for the weak.” These kinds of attitudes, coupled with a lack of understanding of how sleep loss alters physiology may prevent cultural change from taking hold. One way that military culture can be changed is through data, as data can focus attention on the magnitude of a problems. To that end, the goal of this paper is to use survey findings from military service members in five English-speaking countries to address: (1) sleep behavior and attitudes, (2) the relationship between sleep behavior and behavioral health, and (3) leadership and its association with sleep and behavioral health in service members.

## Overview of Studies

The results from the five countries summarized here are an outcome of a technical panel operating under the auspices of The Technical Cooperation Program (TTCP). TTCP is a collaboration involving Australia (AU), Canada (CA), New Zealand (NZ), the United Kingdom (UK), and the United States (US). Given shared interest in understanding sleep, individual countries administered surveys addressing the topic. The surveys were conducted independently and as part of unrelated assessment efforts led by each country, and therefore there was diversity in how these constructs are measured. Nevertheless, the surveys generally included questions about sleep behavior and attitudes, behavioral health, and the role of leadership. Specific questions and the context of these surveys are described below.

### Australia

Data from Australia were collected from members of the Australian Defence Force (ADF), including members of the Australian Army (85.6%), Royal Australian Navy (12.4%), and Royal Australian Air Force (1.8%). These data were collected during Operation Bushfire Assist (OP BA) from 2019 to 2020, during which the ADF joined with firefighters and other emergency responders to provide disaster response and humanitarian assistance for the Australian bushfires. There were 6704 respondents to this survey.

### Canada

Canadian data were collected as part of the Unit Morale Profile survey from three units which were located in garrison (i.e., on a military installation). Unit types ranged from joint staff to operational but contained members from all branches of the Canadian Armed Forces. Data were collected in 2019–2020. In all, there were 739 respondents sampled.

### New Zealand

New Zealand data were collected from 2679 respondents who were members of New Zealand’s fulltime force. The New Zealand Defence Force Health and Wellbeing survey was open to approximately 9000 individuals in 2019.

### UK

Data from the UK were collected from small, deployed units of British Army troops that were engaged in land operations in Afghanistan in 2014, Sudan in 2017, and Special Forces operations in 2020. Activities in which troops were engaged were diverse, including combat, peacekeeping, and surveillance. There were 4520 respondents.

### United States

US data were collected from Army soldiers as part of a larger program evaluation survey effort in 2014. Data are from 2528 respondents who were part of one brigade in garrison.

## Measures

Surveys from each country were comprised of a wide range of questions that varied depending on the study purpose and specific population being surveyed. We focus on relevant survey questions that were similar across countries. Specifically, three of the countries (Canada, New Zealand, and the US) had an item assessing average daily duration of sleep, with 5–6 response options (e.g., 3 h or fewer, 4 h, 5 h, 6 h, 7 h, 8 h, or more). All five countries included questions taken from the Insomnia Severity Index (ISI), a seven-question scale designed to assess the severity of both nighttime and daytime components of insomnia [18]. Due to individual survey requirements, there was variability in terms of which questions were included, so we focused this paper on responses to four of the ISI questions in order to encompass the most countries. Below, we examine responses to the first two ISI questions, which asked participants to rate their “difficulty falling asleep” and “difficulty staying asleep” over the past two weeks. Items were rated using 5 response options from *None* to *Very Severe*. We also examine the ISI question that asked how satisfied/dissatisfied respondents were with their current sleep pattern. Items were rated using 5 response options from Very Satisfied to Very Dissatisfied. Finally, we review responses to the ISI question that asked to what extent respondents considered their sleep problem interfered with daily functioning. This item was rated on a 5-point scale from Not at All/No Sleep Problem to Very Much Interfering. Australia provided answers to the first two ISI questions (see Table 1); Canada, New Zealand, the UK, and the US provided answers to all four questions.

**Table 1:**
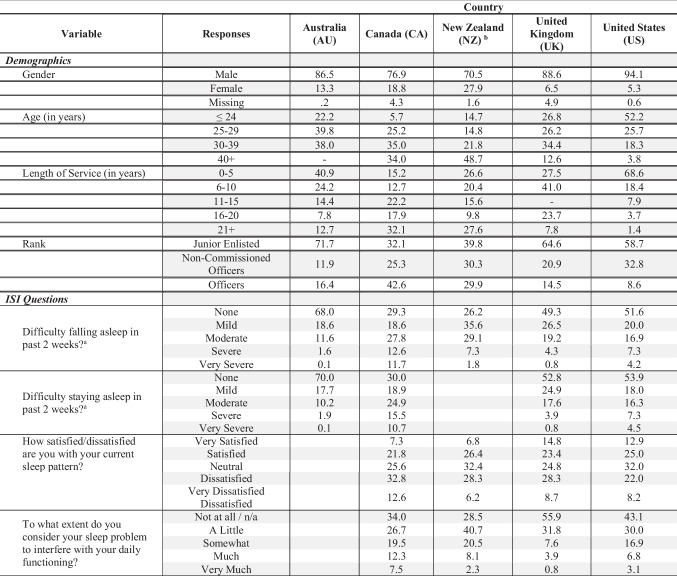
Demographics and insomnia-related questions by country

All numbers represent the percentage of respondents that answered with each response. ISI refers to the Insomnia Severity Index

^*^Age ranges shown follow data from New Zealand (NZ), the United Kingdom (UK), and the United States. Data from Canada are binned as ≤ 24, 25–34, 35–44, and 45 + , and Australia are binned as ≤ 24, 25–34, and 35 +

^**^The UK dataset reported years of service in ranges of 0–4, 5–12, 13–22, and over 22. Percentages are presented in best-fit cells

^a^Questions were asked using the following prompt: “Please rate the current severity of any sleep problems you may have experienced in the past 2 weeks”

^b^The NZ survey combined sleep questions on falling and staying asleep into one item: “During the past two weeks, how much difficulty have you had falling asleep and staying asleep”

Countries varied in how they assessed behavioral health. Psychological distress was measured by Australia, Canada, and New Zealand using the 10-item Kessler Psychological Distress Scale (K10) [19], by the UK using the 12-item General Health Questionnaire (GHQ-12) [20], and the US using the 9-item Patient Health Questionnaire (PHQ-9) [21] and 7-item Generalized Anxiety Disorder scales (GAD-7) [22]. Trauma was measured by Australia and the US using the 17-item Post Traumatic Stress Disorder Checklist for Civilians (PCL-C) [23] and by New Zealand using the 5-item PCL (PCL-5) [24]. Stress was measured by New Zealand using a 25-item life stress measure (e.g., workload, finances, conflict with others) rated on a 5-point scale from Not at All to A Great Degree. In the US, stress was measured using one item about “currently experiencing a stress, emotional, alcohol or family problem” answered as Yes or No [25, 26]. Suicidality was measured by New Zealand using six Yes or No items adapted from the Suicidal Scale of the Mini-International Neuropsychiatric Interview (MINI) [27]. Alcohol misuse was measured by New Zealand and the US using the 3-item Alcohol Use Disorders Identification Test-Consumption (AUDIT-C) [28].

Sleep leadership and general leadership were also assessed by a few of the participating countries. Canada and the US used the 10-item Sleep Leadership Scale [29]. Respondents rated how often their first-line leaders encouraged healthy sleep behaviors, educated unit members about the importance of sleep, modeled good sleep behaviors, promoted an adequate sleep environment, and prioritized and planned for healthy sleep. Items were rated using a 5-point scale from Never to Always. With regard to general leadership, questions varied across countries and addressed leader behaviors consistent with effective leadership. Items were rated on a 5-point scale from Never to Always. General leadership was measured by Canada using items that assessed how often leaders communicate values and visions clearly, support and encourage staff and their development, and instill pride and respect in others. US and UK items [30] assessed how often leaders tell soldiers when they have done a good job, embarrass soldiers in front of others, or try to impress higher-ups by assigning extra work to soldiers.

## Summary of General Demographics

Table 1 summarizes the demographics of respondents to the surveys across the countries. Participants were predominantly male (all datasets > 70% male), reflective of the general demographic makeup of the military in each country. Data from Australia and the UK were relatively evenly distributed across various age groups, while respondents from Canada and New Zealand were relatively older (i.e., 30 or older), with nearly half of the New Zealand sample reporting they were 40 years old or older. In contrast, the US participants were relatively young, with half reporting they were 18–24 years old. With regard to years served and rank, participants from Australia and the UK reported relatively fewer years of service and being junior in rank (71.7% from Australia and 64.6% from the UK were in junior ranks). Canada and New Zealand participants were relatively evenly distributed in terms of years of service and rank while 68.6% of respondents from the US had served from 0 to 5 years and most were junior in rank (E1-E4). These demographic findings are internally consistent, with younger samples having fewer years of service and being younger in rank.

## Sleep Behavior and Attitudes Across Countries

Subjective reports of the average daily duration of sleep were assessed by New Zealand, Canada, and the US. Approximately 1 in 3 respondents from New Zealand (34.9%), 2 in 3 from Canada (67.2%), and more than 3 in 4 US respondents (77.2%) self-reported obtaining less than the recommended 7 or more hours of sleep per night. Importantly, in the US sample, more than 20% reported averaging 4 h or fewer of sleep a day. We note that the populations surveyed may in part account for overall differences across the three countries, with Canada and the US surveying operational units in garrison, while those in New Zealand included a broader sample across the fulltime force. Nevertheless, a substantial number of participants surveyed in all three countries reported insufficient sleep.

Beyond self-reported hours of sleep, surveys also included measures of sleep quality from the ISI (Table 1). The first two ISI questions asked participants to rate their difficulty falling asleep and staying asleep. Approximately 2 in 3 (64.7%) respondents from New Zealand indicated having mild or moderate difficulty falling and staying asleep, while 9.1% had severe or very severe difficulty. A little less than half of Canadian and UK participants responded that they had mild or moderate difficulty falling and staying asleep. Importantly, an additional quarter of respondents from Canada indicated severe or very severe difficulty both falling and staying asleep whereas only about 5% of UK participants endorsed these options. The service members from the US and Australia had relatively lower percentages indicating mild to moderate difficulty falling asleep (30.2% for Australia and 36.9% for the US) and staying asleep (27.9% for Australia and 34.2% for the US).

One question to consider when examining the pattern of these results is how to interpret responses indicating less difficulty with falling and staying asleep. For individuals reporting adequate sleep hours, not having difficulty falling and staying asleep could be a sign of healthy sleep. In contrast, for individuals reporting inadequate sleep hours, these same responses could be seen as a signal of heightened homeostatic sleep pressure; in other words, they are so exhausted that they are able to immediately fall asleep and stay asleep. These patterns can be observed in datasets from Canada, New Zealand, and the US, all of which included measures of sleep quality and average number of hours of sleep. In the US data, over 50% indicated no difficulty falling or staying asleep, compared to approximately 25–30% from Canada and New Zealand. At the same time, most US soldiers reported inadequate sleep, suggesting that their homeostatic sleep pressure may have obviated issues initiating and maintaining sleep. Conversely, respondents from New Zealand were relatively better rested and therefore presumably had less sleep debt, allowing for difficulties to emerge in terms of falling and staying asleep. While sleep problems are normally characterized by difficulty falling and staying asleep, it is critical to also take into account how sleep deprived an individual is before interpreting such measures. Although we do not have data from Australia regarding average sleep duration, respondents were experiencing high operational demands during the bushfire crisis, and thus presumably they had restricted sleep opportunities. Given that approximately 70% of these participants indicated no sleep difficulties in terms of falling or staying asleep, one possible interpretation of these results is that their homeostatic pressure to sleep was so high that it facilitated sleep.

Surveys then asked participants to rate their satisfaction with their sleeping pattern. Four countries had data related to this question, and approximately a quarter (22–26%) indicated they were satisfied. Responses to the questions about difficulty falling and staying asleep were generally consistent with the pattern of responses to the question about satisfaction. The countries that generally reported having *little* difficulty falling and staying asleep (likely because of their high sleep drive), also had relatively less dissatisfaction with their sleep patterns (37.0% for the UK, and 30.2% for the US). In contrast, the one country (Canada) that had comparatively more difficulty falling and staying asleep was relatively dissatisfied with their sleep pattern (45.4%). Essentially, if service members perceive that they are getting quality sleep (i.e., less trouble falling and staying asleep), then they are likely to judge their sleep behavior as satisfactory, and vice versa, regardless of how much sleep they actually obtain. Despite inadequate sleep, many respondents reported that they perceived their daily functioning to not be negatively impacted by their sleep problems (see bottom row of Table 1). This is an important point because it is consistent with sleep research that demonstrates individuals are notoriously bad at estimating the degree to which their lack of sleep is impacting their mood and ability to function; individuals tend to overestimate their resilience to the effects of sleep restriction [31, 32]. Indeed, participants that were asked about their sleep problems impacting their functioning reported little to no interference.

Collectively, these findings confirm that service members are overwhelmingly experiencing challenges with quantity and quality of sleep and are likely to have difficulty estimating the impact of sleep restriction on their performance and health. These problems highlight the potential benefit of educating service members on cost to health and performance associated with sleep restriction. This education could be enhanced through implementation of real-time feedback about sleep behavior and feedback on risk assessed by a wearable device and personalized performance data [33]. Providing this individualized feedback may prove to be less in cost and greater in value than the cost of retaining sleep-deprived service members, though this is an area for future research. These data also suggest that leaders who ask unit members about their sleep should focus not only on the number of hours slept but also on perceived sleep quality and sleepiness, given that all have been associated with health and performance. Educating both individuals and leaders at all levels using personalized findings may help change military culture around sleep.

## Relationships Between Sleep Behavior and Behavioral Health

Numerous previous studies have documented the negative effects of restricted sleep on health outcomes including physiological health conditions, such as increased risk of cardiovascular disease, diabetes, hypertension, obesity, reduced neurocognitive functioning [3, 11, 12, 34], and behavioral health [35]. Critically, insufficient sleep and sleep dysfunction are inextricably related to behavioral health disorders, like depression [36], anxiety [37], and PTSD [38]. While sleep loss and sleep dysfunction are symptoms of many behavioral health disorders, they are also risk factors for developing a behavioral health problem [39, 40] and known to exacerbate the severity of a behavioral health problem. For example, chronic insomnia has been causally linked to the development and exacerbation of depression [39, 40].

The data from all five countries underscore this point. While the surveys varied in terms of how behavioral health was measured and what sample was surveyed, there was a consistently significant and meaningful correlation between sleep problems and behavioral health symptoms. Table 2 summarizes the correlations between responses to ISI questions (either individual questions or as a total score) and behavioral health symptoms.

**Table 2:**
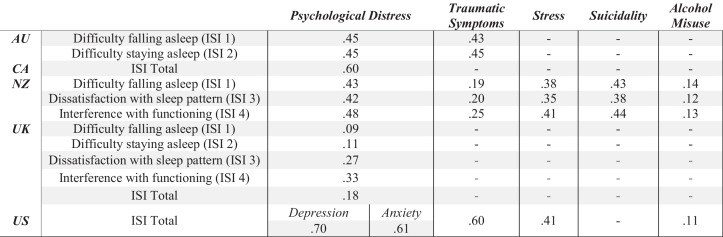
Zero-order correlation between sleep quality and behavioral health symptom across 5 countries

All correlational values are significant at *p* < .001. A dash (-) indicates no data provided to correlate

Sleep was measured using items from the Insomnia Severity Index (ISI). Psychological distress was measured using the 10-item Kessler Psychological Distress Scale (K-10; Australia [AU], Canada [CA], and New Zealand [NZ]), the 12-item General Health Questionnaire (GHQ-12; the United Kingdom [UK]), and the 9-item Patient Health Questionnaire (PHQ-9) and 7-item Generalized Anxiety Disorder (GAD-7) scales (the United States [US])

Trauma was measured using the Post Traumatic Stress Disorder Checklist (PCL) for Civilians (PCL-C; AU), the PCL-5 (NZ), and the PCL (US)

Stress was measured using a 25-item life stress measure (NZ) and a one item stress question (US)

Suicidality was measured using items adapted from the Suicidal Scale of the Mini-International Neuropsychiatric Interview (NZ)

Alcohol misuse was measured using the 3-item Alcohol Use Disorders Identification Test-Consumption (AUDIT-C; NZ and the US)

Australia, Canada, and New Zealand used the K10, a measure assessing depression and anxiety symptoms. In each case, responses on the ISI questions were significantly and positively correlated with psychological distress (all *r* ≥ .42, *p* < .001). These findings indicate that the more individuals reported problems with sleep, dissatisfaction with sleep, and sleep problems interfering with daily life, the more they reported symptoms of psychological distress. The US found similar relationships, using the PHQ-9 measure of depression, and the GAD-7 measure of anxiety. Participant scores on the ISI questions were positively related to both depression (*r*_2480_ = .70, *p* < .001) and anxiety (*r*_2477_ = .61, *p* < .001) symptoms. The UK survey included the GHQ-12, which measures psychological distress. Again, responses to this questionnaire were significantly related to ISI responses (*r*_4312_ = .18, *p* < .001), indicating that having more sleep problems was related to more behavioral health symptoms associated with depression and anxiety. There was also a correlation between sleep problems and PTSD symptoms in Australia, New Zealand, and the US; sleep problems and suicidality in New Zealand; and sleep problems and alcohol misuse in New Zealand and the US. In each case, there was a significant positive relationship between sleep problems and these behavioral health constructs.

Besides these specific behavioral health concerns, New Zealand and the US also measured symptoms of overall stress. In both datasets, overall stress was correlated with more sleep problems. Other research has found that sleep and stress have a cyclical relationship in that the stress response to internal and external stressors can lead to heightened arousal and cognitive rumination [41], which can then make it difficult to fall and stay asleep [42]. Poor sleep, in turn, leads to increased activity in the emotional center of the brain, the amygdala, which results in an increased stress response and decreased ability to regulate emotions [43]. This then feeds back into the cycle, with heightened daytime stress and subsequently disrupted sleep.

While these survey findings are correlational, these patterns reflect this feedback loop and are consistent with other evidence that sleep is a crucial predictor of behavioral health. Furthermore, there is evidence demonstrating that addressing sleep issues may improve behavioral health symptoms [44]. These results point to the importance of addressing sleep not only to optimize performance, but for the health and wellness of service members as well.

## Sleep Leadership

Given the cultural factors that may counter the promotion of healthy sleep in military settings, leaders play a critically important role in mitigating the risk of inadequate sleep. Numerous studies have documented the importance of leaders in supporting health and performance [45, 46], but new studies are emerging that demonstrate the specific impact leadership in the military can have on sleep in service members [47, 48]. By modeling healthy sleep behaviors, educating unit members on the importance of sleep, promoting healthy sleep behaviors, and encouraging a culture of caring for one another, leaders can help mitigate the risk of inadequate sleep. For example, in a group randomized trial, platoon leadership teams who received 1 h of training in the value of sleep and behaviors that promote sleep had more unit members report adequate sleep weeks later relative to a wait-list control group [49••]. Furthermore, when leaders failed to model good sleep behavior, their units are likely to perform poorly on tasks, suggesting insufficient sleep not only affects the individual but the group’s functioning as well [50••].

Within the survey data discussed here, Canada and the US included specific questions about sleep leadership [29]. For both Canadian and US respondents, service members who rated their first-line leaders high on this measure of sleep leadership reported sleeping more (*r*_305_ = .12, *p* = .04 and *r*_2416_ = .19, *p* < .001, respectively) and had lower scores on the ISI, indicating fewer sleep problems (*r*_309_ =  − .15, *p* = .01 and *r*_2434_ =  − .26, *p* < .001, respectively).

While not specific to behaviors associated with promoting sleep, Canadian respondents also rated their leaders on how often they communicated values and visions clearly, supported and encouraged staff and their development, and instilled pride and respect in others. Having leaders who exhibited more of these behaviors was also related to service members having fewer sleep problems (*r*_314_ =  − .26, *p* < .001). Furthermore, the Canadian data revealed that both better sleep leadership and general leadership were correlated with less psychological distress (*r*_640_ =  − .13, *p* = .001; *r*_312_ =  − .27, *p* < .001, respectively), further demonstrating the interconnectedness between sleep and behavioral health outcomes.

Ratings of general leadership qualities [30] were associated with fewer sleep problems in the US sample as well both in terms of platoon leadership at the officer (*r*_2404_ =  − .14, *p* < .001) and non-commissioned officer level (*r*_2435_ =  − .14, *p* < .001). In addition, platoon and non-commissioned officer leadership were inversely correlated with depression (*r*_2381_ =  − .16, *p* < .001; *r*_2410_ =  − 0.21, *p* < .001, respectively) and anxiety (*r*_2379_ =  − .13, *p* < .001; *r*_2408_ =  − 0.19, *p* < .001, respectively). Such results suggest that general leadership qualities of both managers (i.e., officers) and first-line supervisors (i.e., non-commissioned officers) are related to the well-being of service members. UK findings also revealed an inverse correlation between sleep problems and positive leadership (*r*_3037_ =  − .17, *p* < .001). While several studies have documented the unique association between sleep leadership and service member sleep health [49••, 51], the correlations across various countries illustrate how leadership, sleep, and mental health are interwoven.

## Limitations

There are several limitations to this review that warrant mention. First, we relied on self-report rather than objective data with regard to variables such as sleep quantity and quality. Ideally, future research will include the use of wearable devices to more accurately and objectively collect data on sleep behavior.

Second, each country conducted their own survey as part of an independent effort. Therefore, there is variability in the specific items and measures across surveys, potentially restricting the degree to which result patterns can be observed across samples. Relatedly, the timing of the surveys differed, with some being administered many years ago and some quite recently. Ideally, future international research will involve a consistent approach to survey content and be conducted within the same timeframe.

Third, there were differences in both population and operational context across countries. Some surveys were military-wide (New Zealand), other surveys focused on service members in garrison (Canada and the US), and others only included deployed units (Australia and the UK). Different operational demands likely influenced the variability in responses to sleep and behavioral health items. Despite these key differences, there were common patterns regarding the prevalence of sleep loss across environments, its consistent relationship to behavioral health, and its association with leadership.

Finally, the surveys did not directly address cultural differences in values, ideas around sleep, or the role of leaders. Future research may benefit from including questions that examine potential cross-cultural differences related to sleep or behavioral health, military sleep culture, and leadership style. In addition, future research should be encouraged to examine attitudes toward sleep and sleep leadership across a range of culturally diverse countries.

## Conclusions

Across five English-speaking countries, survey data revealed that insufficient sleep and difficulties obtaining healthy sleep are prevalent, and there were consistently strong relationships between sleep challenges and behavioral health problems. These findings demonstrate the universal nature of a problem that is associated with negative health and performance outcomes of service members. Nevertheless, these data offer a promising mitigation strategy to address sleep loss: leader promotion of healthy sleep.

Recent studies have demonstrated that educating leaders in sleep hygiene has a positive impact on the health of subordinates [46, 49••]. In order for leaders to be effective advocates for healthy sleep, it is important that they understand the risks to readiness associated with insufficient sleep, appreciate that sleep loss can be prompted by both external causes (e.g., schedule and environment) and internal causes (e.g., stress), and know practical strategies for addressing periods of anticipated sleep loss. For example, leaders can talk with their unit members about appropriate use of caffeine and light [52•]. Leaders can also implement strategies to improve the sleep environment, such as issuing eye masks, installing blackout curtains, and encouraging the use of earplugs to block noise when appropriate [53]. In addition, leaders can ensure that their unit members are provided with tools for emotion regulation and stress management in order to reduce difficulties in falling and staying asleep.

Moreover, leaders and their units may benefit from introducing methods for maximizing sleep opportunities. Specifically, leaders can be educated in the concept of sleep banking (a form of sleep extension), which entails intentionally getting more sleep (e.g., 9 or more hours). Sleep banking can be particularly useful prior to anticipated sleep loss, such as during the pre-mission phase. Leaders can strategically encourage sleep banking to help pay down sleep debt, as a method for potentially reducing the risk of performance declines during wakefulness [54], improving emotion regulation, and supporting cognitive decision-making [55]. Another potentially useful strategy for leaders to support in their units is tactical napping. The intentional use of naps has been associated not only with an increase in the total amount of sleep obtained but with improvements in alertness, mood, memory, and decision-making [7, 56, 57]. Leaders can also be educated in strategies that have been shown to speed recovery after sleep loss following continuous operations, such as sleep extension and adjustments to their unit’s schedule to accommodate tactical naps. By being aware of their unit members’ sleep patterns, leaders can also make scheduling decisions that support service member health and enable optimal functioning.

By making sleep a priority, and asking service members about their sleep, leaders at all levels can help shift military culture to one where the power of sleep is appreciated. Psychiatrists and other mental health providers are critical in supporting leader education in this topic. If leaders are educated about sleep early in their military career, they can also practice using their knowledge in garrison and training contexts and be better prepared for optimally managing sleep in operational settings. These issues are not limited to one particular military but are relevant for each, even if there are unique country-specific differences that might influence sleep culture. Ensuring a consistent and shared message about the importance of sleep in each of these countries will help support mutual reliance and interoperability.

## Declarations

### Disclaimer

This work was performed and approved for publication under The Technical Cooperation Program (TTCP), a five-country (Australia, Canada, New Zealand, the UK, and the United States) defense research effort. All authors are employees of military armed forces or affiliated organizations. All authors received clearance by their employers to submit the paper for publication; however, the views expressed in this article are those of the authors and do not reflect the official policy or position of any institution with which the authors are affiliated.

## References

[1] Hirshkowitz M, Whiton K, Albert SM, Alessi C, Bruni O, DonCarlos L, Ware JC (2015). National Sleep Foundation’s updated sleep duration recommendations. Sleep Health.

[2] Harrison Y, Horne JA (2000). The impact of sleep deprivation on decision making: a review. J Exp Psychol Appl.

[3] Killgore WD (2010). Effects of sleep deprivation on cognition. Prog Brain Res.

[4] Rasch B, Born J. About sleep's role in memory. Physiol Rev. 2013 Apr;93(2):681–766. 10.1152/physrev.00032.2012. PMID: 23589831; PMCID: PMC3768102. 10.1152/physrev.00032.2012 PMC3768102 23589831

[5] Goldstein-Piekarski AN, Greer SM, Saletin JM, Walker MP (2015). Sleep deprivation impairs the human central and peripheral nervous system discrimination of social threat. J Neurosci.

[6] Holding BC, Sundelin T, Lekander M, Axelsson J (2019). Sleep deprivation and its effects on communication during individual and collaborative tasks. Sci Rep.

[7] Alger SE, Payne JD (2018). Sleep and memory. Stevens’ Handbook of experimental psychology and cognitive neuroscience, Learning and Memory.

[8] Grier T, Dinkeloo E, Reynolds M, Jones BH (2020). Sleep duration and musculoskeletal injury incidence in physically active men and women: a study of US Army Special Operation Forces soldiers. Sleep Health.

[9] Simpson NS, Gibbs EL, Matheson GO (2017). Optimizing sleep to maximize performance: implications and recommendations for elite athletes. Scand J Med Sci Sports.

[10] Mantua J, Naylor JA, Ritland BM, Mickelson C, Bessey A. Sleep loss during military training reduces testosterone in US. army rangers: a two-study series. Int J Sports Exerc Med. 2020;6:169. 10.23937/2469-5718/1510169.

[11] Van Cauter E, Spiegel K, Tasali E, Leproult R (2008). Metabolic consequences of sleep and sleep loss. Sleep Med.

[12] Mullington JM, Haack M, Toth M, Serrador JM, Meier-Ewert HK (2009). Cardiovascular, inflammatory, and metabolic consequences of sleep deprivation. Prog Cardiovasc Dis.

[13] Yoo SS, Gujar N, Hu P, Jolesz FA, Walker MP (2007). The human emotional brain without sleep—a prefrontal amygdala disconnect. Current biology.

[14] Gujar N, Yoo SS, Hu P, Walker MP (2011). Sleep deprivation amplifies reactivity of brain reward networks, biasing the appraisal of positive emotional experiences. J Neurosci.

[15] Palmer CA, Alfano CA (2017). Sleep and emotion regulation: an organizing, integrative review. Sleep Med Rev.

[16] Goldschmied JR (2019). How sleep shapes emotion regulation. Sleep, personality, and social behavior.

[17] Caravalho J (2015). Improving soldier health and performance by moving army medicine toward a system for health. The Journal of Strength & Conditioning Research.

[18] Morin CM, Belleville G, Bélanger L, Ivers H (2011). The Insomnia Severity Index: psychometric indicators to detect insomnia cases and evaluate treatment response. Sleep.

[19] Kessler RC, Andrews G, Colpe LJ, Hiripi E, Mroczek DK, Normand SL, Zaslavsky AM (2002). Short screening scales to monitor population prevalences and trends in non-specific psychological distress. Psychol Med.

[20] Goldberg DP, Gater R, Sartorius N, Ustun TB, Piccinelli M, Gureje O, Rutter C (1997). The validity of two versions of the GHQ in the WHO study of mental illness in general health care. Psychol Med.

[21] Kroenke K, Spitzer RL, Williams JB (2001). The PHQ-9: validity of a brief depression severity measure. J Gen Intern Med.

[22] Spitzer RL, Kroenke K, Williams JB, Löwe B (2006). A brief measure for assessing generalized anxiety disorder: the GAD-7. Arch Intern Med.

[23] Weathers FW, Litz BT, Herman DS, Huska JA, Keane TM. The PTSD Checklist (PCL): reliability, validity, and diagnostic utility. Presented at the annual convention of the International Society for Traumatic Stress Studies, San Antonio, TX. 1993(462).

[24] Blevins CA, Weathers FW, Davis MT, Witte TK, Domino JL (2015). The posttraumatic stress disorder checklist for DSM-5 (PCL-5): development and initial psychometric evaluation. J Trauma Stress.

[25] Hoge CW, Castro CA, Messer SC, McGurk D, Cotting DI, Koffman RL (2004). Combat duty in Iraq and Afghanistan, mental health problems, and barriers to care. N Engl J Med.

[26] Britt TW, Wilson CA, Sawhney G, Black KJ (2020). Perceived unit climate of support for mental health as a predictor of stigma, beliefs about treatment, and help-seeking behaviors among military personnel. Psychol Serv.

[27] Lecrubier Y, Sheehan DV, Weiller E, Amorim P, Bonora I, Sheehan KH, Dunbar GC (1997). The Mini International Neuropsychiatric Interview (MINI). A short diagnostic structured interview: reliability and validity according to the CIDI. Eur Psychiatry.

[28] Bush, K., Kivlahan, D. R., McDonell, M. B., Fihn, S. D., Bradley, K. A., & Ambulatory Care Quality Improvement Project (ACQUIP (1998). The AUDIT alcohol consumption questions (AUDIT-C): an effective brief screening test for problem drinking. Arch Intern Med.

[29] Gunia BC, Sipos ML, LoPresti M, Adler AB (2015). Sleep leadership in high-risk occupations: an investigation of soldiers on peacekeeping and combat missions. Mil Psychol.

[30] Ragins BR (1989). Power and gender congruency effects in evaluations of male and female managers. J Manag.

[31] Balkin TJ, Rupp T, Picchioni D, Wesensten NJ (2008). Sleep loss and sleepiness: current issues. Chest.

[32] Smith CD, Cooper AD, Merullo DJ, Cohen BS, Heaton KJ, Claro PJ, Smith T (2019). Sleep restriction and cognitive load affect performance on a simulated marksmanship task. J Sleep Res.

[33] Adler AB, Gunia BC, Bliese PD, Kim PY, LoPresti ML (2017). Using actigraphy feedback to improve sleep in soldiers: an exploratory trial. Sleep Health.

[34] Goel N, Rao h, Durmer JS, Dinges DF (2009). Neurocognitive consequences of sleep deprivation. Seminars in neurology.

[35] Freeman D, Sheaves B, Waite F, Harvey AG, Harrison PJ (2020). Sleep disturbance and psychiatric disorders. The Lancet Psychiatry.

[36] Tsuno N, Besset A, Ritchie K (2005). Sleep and depression. J Clin Psychiatry.

[37] Alvaro PK, Roberts RM, Harris JK (2013). A systematic review assessing bidirectionality between sleep disturbances, anxiety, and depression. Sleep.

[38] Harvey AG, Jones C, Schmidt DA (2003). Sleep and posttraumatic stress disorder: a review. Clin Psychol Rev.

[39] Staner L (2010). Comorbidity of insomnia and depression. Sleep Med Rev.

[40] Gehrman P, Seelig AD, Jacobson IG, Boyko EJ, Hooper TI, Gackstetter GD. Millennium Cohort Study Team. Predeployment sleep duration and insomnia symptoms as risk factors for new-onset mental health disorders following military deployment. Sleep. 2013;36(7):1009–18. 10.5665/sleep.2798 PMC3669076 23814337

[41] Guastella AJ, Moulds ML (2007). The impact of rumination on sleep quality following a stressful life event. Personality Individ Differ.

[42] Kalmbach DA, Anderson JR, Drake CL (2018). The impact of stress on sleep: pathogenic sleep reactivity as a vulnerability to insomnia and circadian disorders. J Sleep Res.

[43] Minkel JD, Banks S, Htaik O, Moreta MC, Jones CW, McGlinchey EL, Dinges DF (2012). Sleep deprivation and stressors: evidence for elevated negative affect in response to mild stressors when sleep deprived. Emotion.

[44] Fang H, Tu S, Sheng J, Shao A (2019). Depression in sleep disturbance: a review on a bidirectional relationship, mechanisms and treatment. J Cell Mol Med.

[45] Inceoglu I, Thomas G, Chu C, Plans D, Gerbasi A (2018). Leadership behavior and employee well-being: an integrated review and a future research agenda. Leadersh Q.

[46] Brossoit RM, Hammer LB, Crain TL, Leslie JJ, Bodner TE, Brockwood KJ (2023). The effects of a Total Worker Health intervention on workplace safety: mediating effects of sleep and supervisor support for sleep. J Occup Health Psychol.

[47] Hammer LB, Brady JM, Brossoit RM, Mohr CD, Bodner TE, Crain TL, Brockwood KJ (2021). Effects of a Total Worker Health® leadership intervention on employee well-being and functional impairment. J Occup Health Psychol.

[48] Sianoja M, Crain TL, Hammer LB, Bodner T, Brockwood KJ, LoPresti M, Shea SA (2020). The relationship between leadership support and employee sleep. J Occup Health Psychol.

[49] Adler AB, Bliese PD, LoPresti ML, McDonald JL, Merrill JC (2021). Sleep leadership in the army: a group randomized trial. Sleep Health.

[50] Teyhen DS, Capaldi VF, Drummond SP, Rhon DI, Barrett AS, Silvernail JL, Boland DM (2021). How sleep can help maximize human potential: the role of leaders. J Sci Med Sport.

[51] Gunia BC, Adler AB, Bliese PD, Sutcliffe KM (2021). How are you sleeping? Leadership Support, Sleep Health, and Work-Relevant Outcomes. Occupational Health Science.

[52] • Good CH, Brager AJ, Capaldi VF, Mysliwiec V. Sleep in the United States military. Neuropsychopharmacology. 2020;45(1):176–91. **This review article discusses the consequences of insufficient sleep and circadian misalignment in service members and proposes targeted countermeasures that military health care providers can implement.** 10.1038/s41386-019-0431-7 PMC6879759 31185484

[53] Mantua J, Ritland BM, Naylor JA, Simonelli G, Mickelson CA, Choynowski JJ, Bessey AF, Sowden WJ, Burke TM, McKeon AB. Physical sleeping environment is related to insomnia risk and measures of readiness in US army special operations soldiers. BMJ Mil Health. 2023 Aug;169(4):316–320. 10.1136/bmjmilitary-2021-001801. Epub 2021 Jul 22. PMID: 34301851. 10.1136/bmjmilitary-2021-001801 34301851

[54] Rupp TL, Wesensten NJ, Bliese PD, Balkin TJ (2009). Banking sleep: realization of benefits during subsequent sleep restriction and recovery. Sleep.

[55] Alger SE, Brager AJ, Balkin TJ, Capaldi VF, Simonelli G (2020). Effect of cognitive load and emotional valence of distractors on performance during sleep extension and subsequent sleep deprivation. Sleep.

[56] McKeon AB, Mantua J, Sowden WJ. Sleep tactics for multidomain operations in US Army military personnel: optimising sleep opportunities to support readiness and lethality. BMJ Mil Health. 2023 May;169(e1):e1–e3. 10.1136/bmjmilitary-2020-001731. Epub 2021 Aug 20. PMID: 34417342. 10.1136/bmjmilitary-2020-001731 34417342

[57] Alger SE, Brager AJ, Capaldi VF (2019). Challenging the stigma of workplace napping. Sleep.

